# International Congress

**Published:** 1890-06

**Authors:** 


### International Congress—Tenth International Medical Congress.

By direction of Count Arco, the German Ambassador in Washington, the Consul General of the German Empire in New York, Mr. A. Feigel, sends the following, with the request that the medical and secular press of the country give it the greatest possible publicity. It will be noticed that this circular contains a great many particulars not contained in those previously printed.

***Conditions and Rules Referring to the International Medical and Scientific Exhibition in Berlin, August, 1890.***

1. The exhibition will be opened 11 A. M., August 2, 1890, and closed promptly August 11, P. M., in the Landesausstelungs Park. The sections of the International Medical Congress will meet in the same place.

Provisions will be made for dark chambers, rooms for experimental purposes, and appropriate demonstrations by experts.

The exhibition is limited to the following objects: New or improved scientific instruments and apparatuses for biological and strictly medical purposes, inclusive of those for photography and spectral analysis applicable to medicine. New pharmacological, chemical and pharmaceutical materials and preparations. New foods. New or improved instruments for operations in medicine, surgery, special branches, electrotherapy, etc. New plans and models of hospitals, convalescent homes and establishments for disinfection and bathing, arrangements for nursing and transport. New hygienic apparatuses. New tables and charts of a statistical nature. Preparations and models. Teaching apparatuses. Medical literature.

Applications are expected before May 15, 1890,[1] and will be received by the Secretary General of the Congress, Dr. Lassar, Berlin, N. W. Karlstrasse 19. They must be marked "Ausstellungsangelenheit," and accompanied with a printed visiting or business card containing name and address.

1Count Arco has made such arrangements that applications coming from the United States will be received until the first of July.

2. Of each application there ought to be two copies. It is requested that it should contain a brief and accurate description fit to be used in the compilation of the catalogue.

3. The decision as to the admission of all or part of the proposed exhibits rests with the special or general boards of organization; they will send an immediate reply.

The cost of every square metre or part of a square metre of floor or table surface is 10 marks, of wall surface 6 marks. Two (2) metres in height of the wall are free to those adjoining the wall. Exhibits in the interior of the hall pay for one half of the size of the walk immediately surrounding, in addition to the space occupied.

5. Tables will be furnished. Cases, shelves, repositories, must be procured by the exhibitors under the supervision of the committee. Electric light, steam power, etc., can be had by special arrangement.

6. Inflammable objects are excluded. Insurance of those admitted will be secured free of cost, if notice of their value has been given.

7. Packing and unpacking is free of expense to foreigners. Great care will be taken, but no reponsibility. Expressage by Messrs. Jacob & Volentine, Berlin O. Holzmarktstrasse 65.

8. The exhibits must be delivered on or before July 20. Foreign goods will be free of duty, but certificates—to accompany the goods —ought to be obtained from the exhibition office, Karlstrasse 19.

---

The Law Concerning Hypnotism in Sweden.—The question whether a hypnotized person who acts at the suggestion of others is responsible for his actions, is considered in Sweden to depend upon the fact whether he knew of the danger to which he exposed himself in being hypnotized, and submitted to it voluntarily. If so, by the law of that country, he is held responsible, otherwise not.

